# The Impact of Adjuvant Radiotherapy Combined With Targeted or Immunotherapy on the Heart in Breast Cancer

**DOI:** 10.1111/1759-7714.70298

**Published:** 2026-05-24

**Authors:** Dongmin Liu, Yanling Yang, Tingting Chen, Haonan Han, Yajing Yuan, Liming Xu

**Affiliations:** ^1^ Department of Radiation Oncology Tianjin Medical University Cancer Institute & Hospital, National Clinical Research Center for Cancer, Tianjin's Clinical Research Center for Cancer, Key Laboratory of Cancer Prevention and Therapy Tianjin Tianjin China; ^2^ Hubei Key Laboratory of Tumor Microenvironment and Immunotherapy, College of Basic Medical Sciences China Three Gorges University Yichang China; ^3^ Department of Anesthesia Tianjin Medical University Cancer Institute & Hospital, National Clinical Research Center for Cancer, Tianjin's Clinical Research Center for Cancer, Key Laboratory of Cancer Prevention and Therapy Tianjin Tianjin China

**Keywords:** breast cancer, cardiotoxicity, immunotherapy, radiotherapy, targeted therapy

## Abstract

With the increasing adoption of combination therapies involving targeted therapy, immunotherapy, and radiotherapy in breast cancer treatment, the potential cardiotoxicity risks have emerged as a major clinical concern. This article reviews key clinical trial evidence from recent years, including studies published up to March 2025, focusing on the short‐term and long‐term cardiovascular impacts of combination therapies, aiming to provide clinically relevant guidance for decision‐making. Current clinical studies indicate relatively low rates of acute cardiac adverse events during the treatment period. However, long‐term follow‐up data reveal that patients receiving high‐dose radiotherapy (cardiac radiation dose ≥ 25 Gy) show a significantly increased 10‐year risk of cardiovascular events. Emerging evidence suggests that cardiotoxicity exhibits delayed onset characteristics and significant interindividual variability. Therefore, future research should prioritize long‐term cardiac monitoring in combined treatment regimens and actively explore effective strategies to mitigate cardiotoxicity risks. Proposed approaches include establishing comprehensive dynamic monitoring systems integrating radiomics and biomarkers, optimizing radiotherapy dosing to minimize cardiac damage, and developing myocardial protective agents, ultimately achieving optimal balance between treatment efficacy and patient safety.

## Graphical abstract

Adjuvant radiotherapy combined with targeted or immunotherapy in breast cancer is associated with delayed and dose‐dependent cardiotoxicity. Long‐term monitoring, cardiac dose optimization, and integrated biomarker–radiomics strategies are essential to mitigate cardiovascular risk while preserving oncologic efficacy.

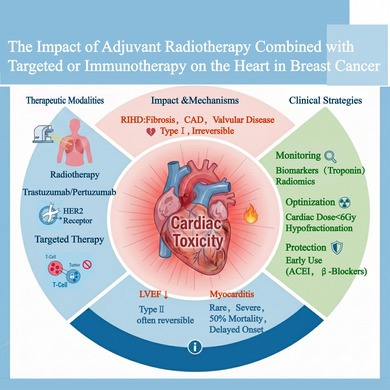

## 1 Introduction

According to the World Health Organization (WHO), while lung cancer remains the malignancy with the highest incidence rate globally, breast cancer incidence has been increasing steadily year by year [1]. Notably, breast cancer has become one of the most rapidly rising cancers in terms of diagnosis rates worldwide. It continues to be a leading cause of cancer‐related mortality among women [2]. However, significant advances in early detection, diagnostic techniques, and therapeutic interventions have progressively improved survival outcomes for breast cancer patients [1, 3]. This progress is largely attributable to the widespread use of innovative therapies, such as targeted treatments, immunotherapy, and radiotherapy, all of which have contributed to a notable and ongoing decrease in breast cancer‐related deaths [4, 5, 6]. However, the proportion of deaths from noncancer‐related causes, particularly cardiovascular diseases, has risen, with cardiovascular conditions now emerging as the leading noncancer‐related cause of mortality in breast cancer survivors [2, 7].

Cancer therapy‐related cardiovascular toxicity represents a major clinical challenge in breast cancer management [3, 8]. This toxicity encompasses a wide range of cardiotoxic effects caused by various cancer treatments, including traditional chemotherapy agents (e.g., anthracyclines, cyclophosphamide, cisplatin, paclitaxel), immunotherapies, and targeted therapies (e.g., trastuzumab, tyrosine kinase inhibitors, vascular endothelial growth factor receptor inhibitors, proteasome inhibitors) [9, 10, 11]. Research suggests that several factors, such as old age, pre‐existing low left ventricular ejection fraction (LVEF), a history of coronary artery disease (CAD), higher cumulative exposure to anthracyclines, the concurrent use of anthracyclines and trastuzumab, and/or radiotherapy, can significantly increase the risk of developing cardiovascular adverse events following cancer treatment [12].

A critical challenge in clinical oncology is achieving an optimal balance between the efficacy of oncological treatments and the preservation of cardiovascular health. As treatment strategies for breast cancer continue to evolve with greater precision, the underlying pathophysiological mechanisms contributing to therapy‐induced cardiotoxicity have proven to be multifaceted and dynamically changing. This review aims to examine the cardiotoxic profiles associated with three cornerstone therapeutic modalities—radiotherapy, targeted therapy, and immunotherapy—and to provide a theoretical framework for the development of cardioprotective strategies in breast cancer management.

## 2 Methods

This article was conducted as a narrative review to summarize current evidence on the cardiovascular effects of adjuvant radiotherapy combined with targeted therapy or immunotherapy in breast cancer.

Relevant literature was identified primarily through a structured search of the PubMed database, supplemented by searches of other commonly used biomedical databases and manual screening of reference lists. Studies published in English from database inception to March 2025 were considered. The search strategy included combinations of keywords such as “breast cancer,” “radiotherapy,” “cardiotoxicity,” “targeted therapy,” and “immunotherapy.”

As a narrative review, no formal risk‐of‐bias assessment was performed. Instead, emphasis was placed on clinically relevant studies, landmark trials, and recent publications. Key themes were developed through narrative synthesis and iterative interpretation of the selected literature, focusing on treatment‐related cardiovascular toxicity, modifying risk factors, and clinical monitoring strategies.

## 3 Current Status of Cardiotoxicity Related to Monotherapy in Breast Cancer

### 3.1 Radiation‐Induced Heart Disease (RIHD)

Radiotherapy is the standard adjuvant treatment for breast cancer and is typically administered after surgery [13, 14]. However, in some cases, it can also be used as the primary treatment or as neoadjuvant therapy for locally advanced tumors [13]. A study conducted in the United States found that breast cancer patients who underwent radiotherapy had a higher proportion of deaths from noncancer causes 5 years posttreatment, compared to those who did not receive radiotherapy. The primary causes of death were heart disease and lung cancer [15]. Taylor et al. [16], through a systematic review of radiation dose distribution characteristics in early‐stage breast cancer, found that the overall mortality rate in patients without breast cancer recurrence increased due to radiotherapy, with most deaths being caused by heart disease. The leading cause was ischemic heart disease, followed by heart failure and valvular heart disease. Over the past few decades, advancements in radiotherapy techniques may have reduced the average radiation dose to the heart, but epidemiological data still suggest that the risk of cardiovascular disease remains unacceptably high [17]. Cardiac adverse effects associated with radiotherapy are commonly referred to as RIHD. Damage may affect any part of the heart, including myocardial fibrosis and cardiomyopathy, CAD, valvular disease, pericardial disease, and arrhythmias. The clinical symptoms are nonspecific, making it challenging to distinguish them in practice.

#### 3.1.1 CAD

Studies have shown that patients who receive left‐sided radiotherapy are at a significantly higher risk of developing CAD compared to those who receive right‐sided radiotherapy [18]. Endothelial injury is a key contributor to CAD, as it triggers an inflammatory response and compromises DNA integrity through oxidative stress. In cancer patients undergoing radiotherapy, there is an increased incidence of lesions in the proximal coronary arteries and sinus openings [19]. Damage to the arterial endothelium can also promote the early development of atherosclerosis, particularly in the left anterior descending artery and right coronary artery [3]. Coronary artery damage typically became apparent approximately 8 years after radiotherapy, with its occurrence influenced by multiple factors, and its progression being accelerated by radiotherapy exposure [20].

#### 3.1.2 Myocardial Disease

Radiation‐induced myocardial injury in breast cancer treatment can be classified into two types: Type I and Type II. Anthracycline drugs and radiation‐induced cardiac toxicity are classified as Type I, which are characterized by irreversible effects with a dose–response relationship. Targeted therapies, such as trastuzumab, can induce Type II cardiac reactions, where heart function may return to normal upon discontinuation of the drug, and there is no dose–response relationship [21]. Accidental radiation exposure to the heart can create an inflammatory and profibrotic environment, leading to endothelial dysfunction, accelerated atherosclerosis, and myocardial fibrosis, which may clinically manifest as CAD or cardiomyopathy [2]. The latent period of myocardial injury could be as long as 10 years, with nonspecific clinical symptoms. By the time of diagnosis, irreversible damage is often already present, and in severe cases, left ventricular diastolic dysfunction and reduced LVEF may occur [18, 22].

#### 3.1.3 Pericardial Disease

As radiation directly affects the pericardium, RIHD is a common manifestation, often considered self‐limiting [20]. Early‐stage symptoms may present as acute pericarditis, while later‐stage manifestations can include pericardial effusion and/or constrictive pericarditis [23]. The average onset time for pericardial disease is about 6 months. Acute pericarditis may develop within days or weeks after radiation exposure, whereas pericardial effusion may appear weeks or months later. Constrictive pericarditis can have a latency period of up to 10 years following radiation therapy [24].

#### 3.1.4 Valvular Heart Disease

Radiation‐induced valvular disease is typically a late manifestation, presenting with symptoms and signs of heart failure due to valve stenosis or regurgitation [25]. Compared to other complications, clinical symptoms of valvular damage are more insidious, with a latency period ranging from 10 to 20 years. The incidence increases when the radiation dose to the heart exceeds 30 Gy [26]. The aortic and mitral valves are most commonly affected, with the risk being related to the radiation dose received, the duration of exposure, and the use of concurrent chemotherapy.

#### 3.1.5 Arrhythmias and Conduction System Disease

Cancer patients undergoing treatment may develop various arrhythmias, including sinus tachycardia, bradyarrhythmias, tachyarrhythmias, and conduction abnormalities, some of which can lead to severe clinical symptoms and even life‐threatening situations [8]. Conduction abnormalities are relatively rare complications, typically caused by direct radiation‐induced damage to the heart's conduction system cells, as well as indirect inflammatory reactions leading to fibrosis. These abnormalities are major contributors to arrhythmias in cancer patients. Right bundle branch block is the most common conduction abnormality observed in cancer patients postradiation therapy, and it may persist for years or even decades after treatment [27].

### 3.2 Targeted Therapy‐Induced Heart Disease

Targeted therapy is a treatment approach that focuses on specific genes or gene products to effectively target and kill tumor cells while minimizing adverse effects on normal tissues [28]. However, some targets in the targeted therapies used for breast cancer overlap with antigens expressed by cells in the cardiovascular system. As a result, targeted therapy may lead to damage to the cardiovascular system, causing cardiovascular adverse events in patients [10].

#### 3.2.1 Trastuzumab‐Induced Heart Disease

In breast cancer patients, 15%–30% exhibit overexpression of the human epidermal growth factor receptor 2 (HER2). HER2‐positive breast cancer patients typically have a poor prognosis, a high risk of recurrence, and a shorter survival time. Trastuzumab is a recombinant monoclonal antibody that targets the HER2 receptor. It has been shown to improve overall survival in HER2‐positive early‐stage breast cancer patients, significantly enhance prognosis, and prolong survival. Clinically, it is often used in combination with docetaxel or paclitaxel [29].

As the first biologic targeted drug for HER2‐positive breast cancer treatment, trastuzumab is associated with several toxic side effects, including asthma, dyspnea, tachycardia, hypotension, and allergic reactions during infusion, with cardiac toxicity being the most severe adverse reaction. Trastuzumab‐related cardiac toxicity typically manifests as an asymptomatic reduction in LVEF, a condition that is often reversible upon discontinuation of the drug [12]. In most cases, LVEF recovered within 1–3 months after treatment completed. However, studies have shown that some cardiotoxic effects of trastuzumab could persist for years (over 30 months) [2].

In a randomized trial, patients were randomly assigned to either the observation group (no trastuzumab treatment) or the experimental group (trastuzumab treatment). After a median follow‐up of 3.6 years, the incidence rates of severe congestive heart failure (CHF) and LVEF reduction were significantly higher in the experimental group, with 0.8% and 3.6%, respectively, compared to the observation group with 0.0% and 0.6%. Most patients in the experimental group who experienced cardiac adverse events had a good prognosis, with a median recovery time of 6.4 months [30]. The OHERA study [31] prospectively analyzed 3700 patients treated with trastuzumab, finding that 106 patients (2.8%) developed symptomatic CHF, and six patients (0.2%) experienced cardiac death. Most of the cardiac adverse events were reversible after treatment. This study suggests that early cardiac toxicity associated with trastuzumab may be reversible.

In clinical practice, it has been observed that when HER2‐targeted therapy is combined with anthracyclines, the cardiac toxicity becomes more pronounced, increasing the risk of heart dysfunction and severe heart failure (5%–10%) [32]. Therefore, during the adjuvant treatment phase for breast cancer, trastuzumab is generally avoided concurrently with anthracyclines. Trastuzumab is typically used after anthracycline therapy or in combination with chemotherapy regimens that do not include anthracyclines. Some studies have also suggested that the use of antioxidants, ACE inhibitors, angiotensin II receptor blockers, beta‐blockers, statins, and other medications in combination with targeted therapy may have a cardioprotective effect [12].

#### 3.2.2 Trastuzumab + Pertuzumab‐Induced Heart Disease

Recent studies reveal 25% disease recurrence in breast cancer patients after 1‐year trastuzumab adjuvant therapy. The combination of pertuzumab (with complementary HER2‐targeting mechanisms) and trastuzumab demonstrates synergistic efficacy and acceptable safety in clinical trials [33]. Table 1 summarizes the impact of dual‐targeted therapy on the heart from various studies.

**TABLE 1:** Comparison of clinical trial results on the impact of dual‐targeted therapy on cardiotoxicity in breast cancer patients.

Author	Year	Median follow‐up time (months)	Sample size (cases)	Treatment regimen	Radiotherapy, *n* (%)	Symptomatic cardiac adverse events	LVEF impairment (%)	LVEF recovery (%)
Baselga et al. [33]	2012	19.3	402 406	T + H + P T + H+ Placebo	No	LVSD: 5 (4.4) vs. 11 (8.3)	≥ Grade 3: 3.8% vs. 6.6%	—
Baselga et al. [34]	2010	—	66	H + P	No	0	3 (0.5)	—
Gianni et al. [35]	2012	19.3	107 107 107 96	T + H T + H + P H + P T + P	No	HF: 0 vs. 0 vs. 1 vs. 0	1 1 3 1	5 (83.3)
Swain et al. [36]	2013	30	408 396	T + H + P T + H+ Placebo	No	LVSD: 5 (1.2) vs. 7 (1.8)	18 (4.6) 28 (7.4)	16 (88.9) 25 (89.3)
Dang et al. [37]	2015	13.8	67	H + P	No	0	1 (1.5)	0
Minckwitz et al. [38]	2017	45.4	2364 2405	T + H + P T + H+ Placebo	No	—	15 (0.6) 6 (0.2)	7 (46.7) 4 (66.7)
Schneeweiss et al. [39]	2013	20–21	225	FEC + H + P × 3 → T + H + P × 3 FEC × 3 → T + H + P × 3 TCH + P × 6	No	LVSD: 0 vs. 1 (0.4) vs. 0	4 (5.9) 4 (5.3) 3 (3.9)	11 (100)
Yu et al. [40]	2016	21	67	T + H + P	No	0	2 (3)	1 (50)
Loibl et al. [41]	2024	101	2404 2400	H H + P	No	HF: 6 (0.2) vs. 16 (0.7) CD: 4 (0.2) vs. 3 (0.1)	6 (0.2) 16 (0.7)	—
Ajgal et al. [42]	2017	12.6	23	T + H + P	Yes	0	1	—
Ben Dhia et al. [43]	2021	38	77	H + P	Yes, breast 41 (53.2); chest wall 29 (37.7)	0	6 (7.8)	5 (83.3)
Aboudaram et al. [44]	2021	49.2	55	H + P	Yes, breast 23; chest wall 30	0	0	—
Yang et al. [45]	2025	11	567 124	H H + P	Yes	0	4 (1.1) vs. 0	—
Dreyfuss et al. [46]	2019	21.4	80	H (60, 75%) H + *P* (16, 20%)	Yes	PE: 2 (2.5) CMP: 5 (6.3)	5 (6.3)	4 (80)

Abbreviations: CD, cardiac death; CMP, cardiomyopath; FEC, 5‐Fluorouracil, Epirubicin, Cyclophosphamide; H, Trastuzumab; HF, heart failure; LVEF, left ventricular ejection fraction; LVSD, left ventricular systolic dysfunction; P, Pertuzumab; PE, pericardial effusion; T, Docetaxel.

Clinical research has indicated that patients receiving the combination therapy of pertuzumab and trastuzumab had average LVEF levels close to baseline or only slightly decreased, with fewer clinical symptoms related to cardiac toxicity [34, 35]. In another trial, 808 patients were randomly assigned to either the pertuzumab group or the placebo group. The results showed that the progression‐free survival (PFS) in the pertuzumab group was extended by 50%, and the proportion of patients who experienced left ventricular systolic dysfunction was higher in the control group (8.3% vs. 4.4%). The control group had 6.6% decreasing in LVEF, while the pertuzumab group had 3.8% [33, 36]. Dang et al. [37] evaluated the efficacy and safety of dual‐targeted therapy with pertuzumab combined with paclitaxel in metastatic HER2‐positive breast cancer patients, finding that the combination had good overall tolerance and no symptomatic left ventricular systolic dysfunction was observed. Minckwitz et al. [38] similarly studied the treatment effect and cardiac safety of dual‐targeted therapy, finding that heart failure, cardiac death, and heart failure‐related events were rare. In the control group, six patients (0.2%) experienced CHF or significant LVEF reduction, whereas in the experimental group, 15 patients (0.6%) had similar issues. Meanwhile, the PEONY study [47] confirmed that dual‐targeted therapy significantly improved treatment outcomes for HER2‐positive breast cancer patients in Asian populations, with safety data consistent with monoclonal antibody therapy. Several other phase II trials also demonstrated that dual‐targeted therapy combined with standard chemotherapy resulted in a lower incidence of symptomatic left ventricular systolic dysfunction [39]. In patients receiving dual‐targeted therapy, no cases of symptomatic heart failure occurred, and only two patients (3.0%) experienced asymptomatic reductions in LVEF [40].

Current data suggest that dual‐targeted therapy not only improves patient prognosis but also does not significantly increase cardiac toxicity compared to monotherapy.

### 3.3 Immunotherapy‐Induced Heart Disease

Immunotherapy has revolutionized the paradigm of cancer treatment and has become one of the essential therapeutic approaches for breast cancer [48]. Triple‐negative breast cancer (TNBC), defined by the absence of estrogen receptor (ER), progesterone receptor (PR), and HER2 expression, is characterized by its aggressive behavior, high recurrence rate, and propensity for metastasis [49]. Due to the lack of actionable therapeutic targets, chemotherapy remains the mainstay treatment for TNBC. However, clinical evidence demonstrated that most TNBC patients exhibited poor responses to chemotherapy, with limited survival benefits and significant treatment‐related toxicities. With advancements in immunotherapy, TNBC is now recognized as the most immunogenic breast cancer subtype, providing novel therapeutic opportunities [50, 51].

Current immunotherapeutic strategies encompass various approaches including adoptive cell transfer therapy, cancer vaccines, oncolytic viruses, and immune checkpoint inhibitors (ICIs). Recent research has predominantly focused on ICIs, particularly programmed death‐1 (PD‐1)/programmed death‐ligand 1 (PD‐L1) inhibitors, which enhanced antitumor immunity by competitively blocking PD‐1/PD‐L1 interaction to restore T‐cell‐mediated tumor killing [52]. Clinically approved agents such as pembrolizumab, atezolizumab, durvalumab, and avelumab have demonstrated significant efficacy in breast cancer treatment through multiple clinical trials [53, 54].

Despite their therapeutic benefits, ICIs are associated with rare but potentially life‐threatening immune‐related adverse events, particularly cardiovascular toxicities. These include myocarditis, pericarditis, arrhythmias, conduction blocks, and new‐onset heart failure, with myocarditis being the most frequently reported manifestation. Although rare, these cardiac complications necessitate careful monitoring, as their pathogenesis is still not fully understood [55]. The timing of cardiotoxicity onset correlates with multiple factors including patient history, specific checkpoint inhibitor type, treatment duration, and whether monotherapy or combination regimens are administered.

In a retrospective study, the incidence of ICI‐related vascular toxicity was 6.3%, with the most common cardiac complications being myocarditis (15%), atrial fibrillation (13%), pericardial disease (13%), heart failure (17%), and CAD (19%). Notably, myocarditis had a mortality rate as high as 50% [56]. Mahmood et al. [57] compared 35 patients with ICI‐related myocarditis to a randomly selected group of 105 ICI‐treated patients without myocarditis. They found that before ICI therapy, most patients had normal LVEF and electrocardiogram (ECG). Myocarditis was the first immune‐related adverse event (irAE) in approximately 50% of cases, and, in contrast to nonimmune‐related myocarditis, a reduction in LVEF was not a prerequisite for severe cardiovascular adverse events.

The risk factors for ICI‐associated myocarditis remain unclear. However, dual ICI therapy, a history of autoimmune disease, pre‐existing cardiac disease, heart failure, acute coronary syndrome, diabetes, and advanced age may increase the risk of myocarditis. Several studies analyzing breast cancer patients treated with pembrolizumab have reported common treatment‐related adverse events such as arthralgia, fatigue, nausea, anemia, and myalgia, but no significant cardiac toxicity has been documented [53, 58, 59].

Currently available anti‐PD‐L1 antibodies exhibit relatively high immunogenicity and are associated with significant side effects. Due to the limited number of relevant studies and small sample sizes, no severe cardiac toxicity related to ICI therapy has been observed in the existing literature (Table 2). However, ICI‐associated myocarditis remains challenging to diagnose due to the lack of specific symptoms, clinical signs, electrocardiographic findings, laboratory markers, and imaging characteristics [63].

**TABLE 2:** Summary of cardiac and noncardiac adverse events associated with immunotherapy.

Author	Year	Median follow‐up time	Sample size (cases)	ICI‐related myocarditis	Death cases (%)	Main adverse reactions (%)	Abnormal indicators (%)
Mahmood et al. [57]	2018	102 days	—	35	—	Chest pain: 13 (37) Dyspnea: 20 (58) Orthopnea: 6 (16) Paroxysmal nocturnal dyspnea: 6 (16) Fatigue: 7 (21)	ECG abnormalities: 31 (89) Troponin I increase: 33 (94) Decrease in LVEF: 17 (49)
Nanda et al. [58]	2016	10 months	32	0	1	Arthralgia: 6 (18.8) Fatigue: 6 (18.8) Myalgia: 6 (18.8) Nausea: 5 (15.6)	—
Adams et al. [59]	2019	9.6 months	170	1 (0.6)	136 (80)	Fatigue: 35 (20.6) Nausea: 19 (11.2) Hypothyroidism: 14 (8.2) Decreased appetite: 13 (7.6) Diarrhea: 12 (7.1) Weakness: 11 (6.5) Itching: 11 (6.5) Arthralgia: 10 (5.9)	——
Schmid et al. [53]	2020	19.6 months	60	0	1 (1.7)	Neutropenia: 53 (88) Nausea: 40 (67) Anemia: 34 (57) Hypothyroidism: 5 (8) Colitis: 3 (5) Skin reaction: 3 (5) Hepatitis1 [2]	——
Voorwerk et al. [60]	2019	19.9 months	70 (12 cases of RT)	0	2 (2.9)	Induction therapy‐related adverse events: 19 (28) Grade 3–5 immune‐related adverse events: 13 (19)	—
Tison et al. [61]	2023	12 months	28 (concurrent RT) 27 (sequential RT)	0 (0) vs. 7 (25.9)	0	Dermatitis: 24 (85.7) vs. 23 (85.1) Fatigue: 15 (53.6) vs. 13 (48.2) Pain: 4 (14.3) vs. 8 (29.6) Dysphagia: 4 (14.3) vs. 3 (11.1) Cough: 4 (14.3) vs. 3 (11.1) Dyspnea: 4 (14.3) vs. 4 (14.8) Lymphedema: 2 (7.1) vs. 3 (11.1)	—
McArthur et al. [62]	2017	13 months	17 (RT)	0	5 (29.4)	Dermatitis: 1 (5.9) Nausea: 2 (11.8) Diarrhea: 2 (11.8) Fatigue: 5 (29.4) Fever: 2 (11.8) Soft tissue infection: 1 (5.9)	——

Abbreviations: CHF, congestive heart failure; LVEF, left ventricular ejection fraction; RT, radiotherapy.

At present, the use of ICIs in breast cancer treatment is relatively limited, primarily being effective in TNBC. As immunotherapy continues to advance rapidly, the indications for its use are expected to expand, enabling more patients to benefit from immune‐based treatments. In clinical practice, more sensitive biomarkers and diagnostic tools will be necessary to facilitate the early detection and prevention of ICI‐associated myocarditis.

## 4 Cardiotoxicity Associated With Combination Therapy for Breast Cancer

### 4.1 Targeted Therapy Combined With Radiotherapy

Radiotherapy for breast cancer is typically administered postoperatively. Given that breast cancer surgery is classified as a low‐risk procedure with a cardiac risk of less than 1%, this section will focus solely on the combination of targeted therapy and radiotherapy. In comprehensive breast cancer treatment, the cardiotoxic effects of radiation‐induced Type I damage, compounded by Type II damage from targeted agents, have become a significant clinical concern [64].

For HER2‐positive breast cancer patients who are candidates for targeted therapy, cardiac function should be evaluated prior to radiotherapy. If cardiac function is normal, targeted therapy can be administered concurrently with radiotherapy. For patients receiving targeted therapy for left‐sided breast cancer, the need for internal mammary node irradiation should be carefully evaluated. When radiotherapy is required, advanced intensity modulated radiotherapy (IMRT), treatment techniques should be employed to minimize cardiac exposure, with the mean heart dose ideally kept below 6 Gy [2].

#### 4.1.1 Trastuzumab Combined With Radiotherapy

Current studies indicate that the combination of anthracyclines and trastuzumab increases the risk of heart failure (HF), while radiotherapy increases the risk of ischemic heart disease, valvular disease, and heart failure. Given the radiosensitizing effect of trastuzumab on breast cancer cells [65], it remains inconclusive whether targeted therapy exacerbates the cardiotoxicity of radiotherapy.

Some researchers have found that trastuzumab combined with postoperative adjuvant therapy for breast cancer results in an acceptable level of early cardiac toxicity (Table 1). Abouegylah et al. [66] categorized patients into left‐sided and right‐sided breast cancer groups and analyzed cardiac toxicity after trastuzumab and radiotherapy.

The study found a statistically significant difference in the incidence of arrhythmias, with rates of 14.2% in left‐sided cases compared with less than 1% in right‐sided cases. Additionally, the proportion of ischemic heart disease was higher in left‐sided patients.

A retrospective study by Andersen et al. [15] also demonstrated that in HER2‐positive breast cancer patients, trastuzumab combined with radiotherapy, with or without anthracycline chemotherapy, led to a persistent decline in LVEF compared to trastuzumab monotherapy. The extent of LVEF reduction was correlated with cumulative radiation dose. However, the degree of LVEF reduction was similar in both left‐sided and right‐sided breast cancer patients. The authors did not follow up on whether the LVEF decline was reversible, nor did they provide detailed statistics on clinical complications.

However, increasing evidence suggests that in breast cancer patients, recent clinical studies have further confirmed that concurrent trastuzumab and adjuvant radiotherapy after surgery does not lead to a higher incidence of acute complications (Table 3).

**TABLE 3:** Comparison of clinical trial results on the cardiotoxic effects of trastuzumab targeted therapy in breast cancer patients.

Author	Year	Median follow‐up (months)	Sample size (cases)	RT, *n* (%)	Symptomatic cardiac adverse events (%)	Cardiac dysfunction leading to targeted therapy discontinuation (%)	LVEF impairment (%)	LVEF recovery (%)	Median LVEF recovery time (months)
Andersen et al. [15]	2021	12	135	77 (57)	—	—	LVEF decline ≥ 10% or LVEF < 50%: 19.1%	—	—
Lidbrink et al. [31]	2019	61	3733	1337 (35.8)	HF: 106 (2.8) CD: 6 (0.2)	—	251 (7.6)	169 (67.3)	8.8
Abouegylah et al. [66]	2019	81 94	106 96	Yes, L‐RT Yes, R‐RT	Arrhythmias: L‐RT: 15 (14.2%) vs. R‐RT: 1 (1%) Myocardial damage (ECG): L‐RT: 10 (9.4%) vs. R‐RT: 1 (1%)	—	LVEF decline ≥ 10%: L‐RT: 23 (21.7) vs. R‐RT: 23 (24)	—	—
Bonzano et al. [67]	2018	60	29 23	Yes, L‐RT Yes, R‐RT	—	—	Grade 1: 9 (31) vs. 4 [18] Grade 2: 1 (3) vs. 2 [8]	—	—
Cao et al. [68]	2015	6.7 (LVEF) 26 (Clinical evaluation)	64 73	Yes, H + RT Yes, only RT	0 0	0 0	Grade 1: 5 (7.8) 3 (4.1)	5 (100) 3 (100)	3.16 3.33
Marinko et al. [69]	2018	57	84 91	Yes, L‐RT Yes, R‐RT	PE: 9 (11) vs. 1 [1]	—	LVEF decline ≥ 10% or LVEF < 50%: 4 (4.8) vs. 5 (5.5)	—	—
Meattini et al. [70]	2014	51.6	95	Yes	Dyspnea due to HF: 1 (1.1)	5	≥ Grade 1: 54 (56.8) ≥ Grade 2: 3 (3.2)	16 (27.6)	—
Halyard et al. [71]	2009	44	1274 (H) 664 (no H)	Yes, 935 (73.4%) No/unknown: 339 (26.6) Yes, 483 (72.7) No/unknown, 181 (27.3)	HF: 15 (1.6) vs. 13 (3.8) CD: 0 (0) vs. 2 (0.6) HF: 1 (0.2) vs. 1 (0.6) CD: 1 (0.2) vs. 0 (0)	—	—	—	—
Huszno et al. [72]	2013	—	120	91 (76)	PE: 2 CA: 2	5 (4)	LVEF decline ≥ 10%: 10 (8)	—	—
De Santis et al. [73]	2018	38	176 (no H) 54 (H)	Yes, Hypo‐RT	MI: 1 (0.6) vs. 0	5 (2.8)	Grade 1: 12 (6.8) vs. 7 (13.7) Grade 2: 2 (1.1) vs. 1 [2] Grade 3: 1 (0.6) vs. 0	—	—
Sayan et al. [74]	2019	36 32	41 100	Yes, Hypo‐RT Yes, Conv‐RT	0	0	3 (7) 5 (5)	0	0
Shaffer et al. [75]	2008	15	59	44 (74.6)	HF: 3 (5.1)	11 (18.6)	Mean decline (MD): 4%	—	—
Caussa et al. [76]	2010	28	106	Yes, IMC 83%	Myocarditis: 1 (1) Pericarditis: 3 (2.8)	3 (3)	≥ Grade 2: 6 (5.6)	—	—
Belkace'mi et al. [77]	2008	16	146	Yes, IMC 70.5%	—	—	≥ Grade 2: 10%	—	—
Horton et al. [78]	2010	38	12	Yes	0	0	1 (8.3)	1 (100)	—

Abbreviations: CA, cardiac arrest; CD, cardiac death; Conv‐RT, conventional radiotherapy; HF, heart failure; H + RT, trastuzumab + radiotherapy; Hypo‐RT, hypofractionated radiotherapy; IMC, internal mammary chain; L‐RT, left‐sided radiotherapy; LVEF, left ventricular ejection fraction; MI, myocardial infarction; PE, pericardial effusion; R‐RT, right‐sided radiotherapy; RT, radiotherapy.

Regarding cardiac safety in left‐sided breast cancer patients, a study by Cao et al. [68] evaluated early cardiac toxicity in patients receiving left‐sided radiotherapy with or without concurrent trastuzumab. The results showed that patients in the trastuzumab plus radiotherapy group exhibited good treatment tolerance, with only 7.8% experiencing Grade 1 LVEF decline, and all cases recovered completely. This conclusion aligns with findings from Marinko et al. [69], who reported no statistically significant differences in early cardiac toxicity parameters between left‐ and right‐sided radiotherapy combined with trastuzumab. However, the incidence of pericardial effusion was relatively higher in the left‐sided treatment group.

For long‐term follow‐up, Meattini et al. [70] conducted a retrospective study involving 95 patients to assess the efficacy and tolerability of concurrent trastuzumab and adjuvant radiotherapy, with a median follow‐up of 4.3 years. In the combination therapy group, 57 patients (60.0%) experienced a reduction in LVEF (Grade 1 in 54 cases and Grade 2 in 3 cases), and 16 out of 57 (28.1%) showed LVEF recovery after discontinuing trastuzumab.

Additionally, Halyard et al. [71] analyzed data from the N9831 randomized trial, which included 1938 breast cancer patients with a median follow‐up of 3.7 years. Among them, 1418 patients (73.2%) received radiotherapy, and 935 (48.2%) underwent trastuzumab plus radiotherapy. The results indicated that concurrent trastuzumab and radiotherapy did not significantly increase the incidence of acute cardiac adverse events. In fact, the rate of cardiac events was lower in the radiotherapy group compared to patients who received no radiotherapy or had an unknown radiotherapy status. Furthermore, among 44 patients who received internal mammary chain (IMC) irradiation, no increase in cardiovascular toxicity was observed.

In another study, only 8.0% of patients receiving combined therapy experienced LVEF reduction, and 4.0% discontinued treatment due to cardiac toxicity. However, due to the relatively short follow‐up period, long‐term cardiac toxicity remains uncertain. Potential risk factors that warrant attention include a history of left‐sided chest radiotherapy, high BMI, hormone receptor negativity, and low baseline LVEF [72].

Impact of radiotherapy dose fractionation on cardiotoxicity: Several studies have provided new insights into the effects of radiotherapy dose fractionation [67]. Monitored patients receiving three‐dimensional conformal radiotherapy using echocardiography and found that only mild to moderate cardiac toxicity occurred across different radiotherapy dose groups. Additionally, no significant difference in LVEF decline was observed between left‐ and right‐sided radiotherapy groups.

Furthermore, De Santis et al. [73] investigated whole‐breast hypofractionated radiotherapy protocols and confirmed that this approach, when combined with trastuzumab, did not increase the risk of cardiac toxicity. However, further research is needed to confirm its protective effects. A retrospective study by Sayan et al. [74] demonstrated that the incidence of acute cardiac toxicity remained low and similar between the hypofractionated radiotherapy group and the conventional fractionation radiotherapy group. However, long‐term follow‐up is required to assess the risk of late‐onset cardiac toxicity.

It was previously believed that routine irradiation of lymph node regions could increase cardiac toxicity, particularly in left‐sided breast cancer patients. However, studies have shown that the combination of trastuzumab and radiotherapy (including IMC) does not increase the risk of acute cardiac toxicity. A retrospective study by Shaffer et al. [75] included 59 patients receiving trastuzumab, among whom 44 underwent adjuvant radiotherapy (including IMC), with 13 receiving left‐sided IMC irradiation. The results showed that 18.6% (11/59) of patients discontinued trastuzumab due to cardiac toxicity; paradoxically, none of these patients had received left‐sided IMC irradiation, suggesting that trastuzumab combined with IMC irradiation did not lead to acute cardiac toxicity. However, the authors emphasized that the sample size was small, and further large‐scale studies are needed to confirm this conclusion. A prospective study by Caussa et al. [76] further supported this finding, showing that among 106 patients receiving the combination treatment, only 6 (5.7%) experienced reversible LVEF reduction, and all cases fully recovered with appropriate interventions, leaving no significant cardiac damage. Another retrospective study analyzed 146 patients, among whom 32 (23%) received weekly trastuzumab and 114 (77%) received trastuzumab every 3 weeks, with 71% undergoing IMC irradiation. The results showed that only 10% of patients experienced a ≥ Grade 2 LVEF decline, and local irradiation (IMC and supraclavicular) was not significantly associated with an increased risk of cardiac toxicity [77]. Overall, current evidence supports the cardiac safety of trastuzumab combined with radiotherapy (including IMC irradiation), but long‐term follow‐up data and larger sample sizes are still needed to further validate these findings.

#### 4.1.2 Trastuzumab and Pertuzumab Combined With Radiotherapy

There is limited literature on the combination of dual‐targeted therapy and radiotherapy, with most studies being retrospective and lacking large‐scale data. Available studies suggest that dual‐targeted therapy combined with radiotherapy does not significantly increase early cardiac toxicity (Table 2).

Ajgal et al. [42] conducted a study on 23 patients who received radiotherapy to the chest region or metastatic sites while maintaining dual‐targeted therapy. Among them, 18 patients underwent local irradiation of the primary tumor area, and no significant decrease in LVEF was observed. One patient experienced asymptomatic LVEF reduction, with no other symptomatic cardiac events reported. BenDhia et al. [43] conducted a retrospective analysis of 77 patients, all of whom had normal cardiac assessments before treatment and received dual‐targeted therapy. Forty‐one patients underwent radiotherapy to the breast, while 29 received radiotherapy to the chest wall. Of these, 66 patients were evaluated for cardiac function after completing radiotherapy. One patient showed asymptomatic LVEF reduction, and after follow‐up, six patients exhibited asymptomatic LVEF decrease, with a median onset of 8 months postradiotherapy. Five of these patients' cardiac function returned to normal after discontinuing targeted consolidation therapy, allowing them to continue treatment. Aboudaram et al. [44] studied 55 breast cancer patients receiving dual‐targeted therapy combined with radiotherapy. No cardiac events occurred during the treatment period. Among patients who received local radiotherapy, LVEF decreased by 2.43% compared to pretreatment levels, but none experienced an LVEF reduction greater than 10% or an LVEF below 55%. No significant difference in LVEF was observed between left‐sided and right‐sided radiotherapy patients, with both groups showing a median LVEF of 65%. Yang et al. [45] studied breast cancer patients receiving postoperative radiotherapy with either single or dual‐targeted therapy, with a median follow‐up of 11 months. Cardiotoxicity was evaluated using ECG, LVEF, left ventricular diastolic dysfunction (LVDD), NT‐proBNP, and Cardiac troponin levels. The results showed that the early cardiac effects of single or dual‐targeted therapy combined with radiotherapy were primarily reflected in a higher incidence of ECG abnormalities (59% vs. 67.7%). The incidence of LVEF reduction and LVDD was lower (1.1% vs. 0%, 8.2% vs. 5.5%). Patients receiving higher doses of cardiac radiation were more likely to experience adverse cardiac effects. Dreyfuss et al. [46] demonstrated that low‐fractionated radiotherapy combined with dual‐targeted therapy in early‐stage breast cancer is both effective and associated with acceptable toxicity levels.

Current literature suggests that breast cancer patients receiving dual‐targeted therapy combined with radiotherapy exhibit good cardiac tolerance. However, since existing studies are still insufficient, the potential risk of cardiac toxicity cannot be entirely excluded.

### 4.2 Immunotherapy Combined With Radiotherapy

Radiotherapy has been shown to exert an immune‐activating effect on the body, with the extent of these effects varying depending on the irradiation dose. High‐dose radiotherapy (> 5 Gy per fraction) can activate multiple immune pathways, thereby modifying the tumor microenvironment and stimulating both innate and adaptive immune responses [79]. Conversely, low‐dose radiotherapy (< 2 Gy per fraction) promotes the infiltration of immune T cells into the tumor region and stimulates tumor angiogenesis, which can enhance the therapeutic efficacy of radiotherapy [80].

Although there are limited reports on the combination of immunotherapy and radiotherapy in breast cancer (Table 3), most studies have focused on basic research. In one murine study, the use of a PD‐1 inhibitor alone induced minimal cardiac toxicity. However, when combined with radiotherapy, the PD‐1 inhibitor increased the infiltration of CD8+ T lymphocytes in the heart, elevated levels of inflammatory cytokines such as IL‐1β, IL‐6, and TNF‐α, and amplified the immune‐inflammatory response, thus exacerbating RIHD. Notably, this inflammatory response was more pronounced in the early phase (1 month) compared to the late phase (3 months). Similarly, another study found that mice receiving both cardiac irradiation and PD‐1 blockade therapy had a higher cardiac mortality rate [81]. In a phase I trial of advanced breast cancer, Voorwerk et al. [60] combined radiotherapy with nivolumab treatment and found no serious adverse events. Tison et al. [61] examined the concurrent administration of pembrolizumab and radiotherapy, and the results showed no significant differences in toxicity between the concurrent and nonconcurrent radiotherapy groups. All ICI‐related myocarditis occurred during neoadjuvant pembrolizumab treatment. In a phase II clinical trial, 17 patients who received pembrolizumab in combination with radiotherapy were evaluated. The results indicated good safety with mild common toxicities, including fatigue, myalgia, and nausea [62].

The synergistic effect between radiotherapy and the immune system is increasingly recognized, yet further preclinical and clinical studies are still required to fully understand the mechanisms involved [82]. As such, the clinical application of immune‐radiotherapy in breast cancer treatment remains uncertain.

## 5 Cardiotoxicity Monitoring and Prevention

There are many methods commonly used in clinical practice to detect cardiotoxicity, including electrocardiogram (ECG), LVEF, cardiac enzyme profiles, troponins, and brain natriuretic peptide (BNP), among others. However, most clinical trials and retrospective analyses still focus on LVEF as the primary observation parameter to analyze changes in cardiac function, using CHF as the endpoint.

ECG is an affordable and easy‐to‐use auxiliary diagnostic tool that is widely employed to assess cardiac toxicity caused by medications and radiotherapy. Changes in ECG indicators may suggest alterations in myocardial conduction and repolarization [83]. Abnormal T waves can indicate early myocardial ischemia, often without clinical symptoms, but they can point to early myocardial injury caused by breast cancer treatment [84].

ECG is a commonly used imaging method for cardiac assessment. Depending on clinical indications, various types of echocardiography are used, including M‐mode, Doppler, two‐dimensional (2D)/three‐dimensional (3D), transthoracic or transesophageal, contrast, or stress echocardiography [85]. Transthoracic echocardiography (TTE) is the first‐line screening method for RIHD. It allows for a comprehensive assessment of heart structure and function, is widely available, repeatable, and does not involve radiation [84]. When TTE fails to provide a diagnosis or requires further improvement, TTE is recommended, provided there are no contraindications [85]. However, since the accuracy of echocardiography is highly dependent on the operator's technical skills and its reproducibility is limited, it may not be sensitive enough to detect cardiotoxicity.

LVEF is also commonly used to detect cardiotoxicity. The anti‐HER2 drug trastuzumab has a certain impact on LVEF. The overall clinical incidence is reported to be between 4.2% and 13.1%. In most cases, changes in LVEF gradually recover after discontinuation of targeted therapy, but typically do not return to baseline levels, and patients often require continued cardiac medication. The damage may persist or even worsen, making LVEF an insufficiently sensitive indicator for measuring cardiotoxicity [75].

Tissue Doppler imaging (TDI) can be used to measure systolic and diastolic velocities and provides more sensitive early changes, even when LVEF remains unchanged, to predict cancer treatment‐related heart failure. However, it is also limited by technical issues [86].

Fluorodeoxyglucose (FDG) uptake can also be used to detect cardiotoxicity. One major advantage of FDG‐PET imaging is its ability to detect early myocardial metabolic changes that occur before a decrease in LVEF [87]. Under physiological conditions of blood flow and oxygen supply, one‐third of the energy provided to the myocardium comes from glucose, while two‐thirds come from aerobic metabolism of fatty acids. If myocardial aerobic respiration is affected, it leads to increased anaerobic fermentation energy supply, resulting in increased glucose and FDG uptake. Therefore, FDG uptake reflects the extent of myocardial cell damage and can be used as an early detection method for radiation‐induced heart disease and other conditions [88].

Biomarkers are another effective means of evaluating and tracking cardiac side effects in patients. By measuring biomarkers in blood samples, signs of cardiac injury can be detected earlier, allowing for timely adjustments to treatment plans and reducing potential cardiac risks [87]. Cardiac troponin T (cTnT) and I (cTnI) are cardiac‐specific structural proteins and are specific biomarkers for myocardial injury. However, since various causes of myocardial injury can lead to elevated cTnT/cTnI levels, careful differentiation is required in clinical use [2]. Studies have shown that patients with elevated troponins when treated with HER2 and/or anthracycline‐based drugs are at a higher risk of left ventricular systolic dysfunction [89]. In the general population, BNP is stored in the ventricular myocardium, and impaired heart function can lead to elevated serum BNP levels, with the extent of elevation positively correlated with the severity of heart failure. Additionally, NT‐proBNP has higher sensitivity in assessing cardiac function [12].

## 6 Discussion

Currently, the treatment of breast cancer primarily involves a comprehensive approach, with different treatment plans chosen for various stages and molecular subtypes of tumors. The diversity of treatment options also complicates the adverse reactions and complications, among which cardiotoxicity is an issue that cannot be ignored during any cancer treatment process. RIHD was once considered a late‐onset side effect that would appear only many years after radiotherapy. However, further research has revealed that it has both subacute and long‐term effects, with the extent of these effects highly dependent on the individual patient's factors and the specific conditions of radiotherapy [84].

In a prospective study conducted by Bostany et al. [7], 829 patients were followed up over a long period. The results showed that the incidence of heart failure was 1.8% after 2 years of treatment, increasing to 8.7% at 10 years, and reaching 15.3% at 15 years. Additionally, patients receiving anthracycline‐based chemotherapy and targeted therapy had a higher risk compared to those who only received left breast radiotherapy (HR = 3.92). Darby et al. [22] also found that women who received radiotherapy on the left breast had a higher likelihood of coronary artery events than those who received radiotherapy on the right breast. The incidence of coronary artery events increased linearly with the average radiation dose to the heart, with each Gy increasing the risk by approximately 7.4%, showing no clear threshold‐any radiation causes damage. This effect was more pronounced in patients with a history of cardiovascular disease, and it could persist for at least 20 years. Another cohort study confirmed the dose–response relationship between the average cardiac dose during radiotherapy in breast cancer patients and the risk of acute cardiovascular events. The study followed 910 patients for up to 9 years, and the results showed that for every 1 Gy increase in the average heart dose, the cumulative incidence of cardiovascular events increased by 16.5% [90].

Although existing studies suggest that targeted therapy/immunotherapy combined with radiotherapy does not significantly increase the risk of early cardiovascular toxicity, we still believe that the related long‐term cardiotoxicity should not be overlooked, particularly in patients who receive high‐dose radiotherapy, have a history of cardiovascular disease, or are undergoing combined treatments.

## 7 Conclusion

As treatment models gradually become more personalized and precise, predicting, monitoring, and managing the risk of cardiotoxicity in breast cancer patients has become increasingly important. Before undergoing treatment, breast cancer patients should undergo a comprehensive evaluation of their heart health, including a detailed medical history, electrocardiogram, and echocardiography. Patients with cardiac risk factors (such as advanced age, obesity, smoking, a history of cardiovascular disease, diabetes, or family history) should be stratified accordingly. During treatment, it is recommended to closely monitor the patient's heart condition using multiple indicators, identify any cardiac adverse events in a timely manner, and adjust the treatment plan based on the patient's cardiac status and tolerance. The treatment should be reconsidered for continuation only after the heart function improves. In addition to the currently used methods for detecting cardiotoxicity, future research and practice should explore new techniques for heart function assessment to detect cardiotoxicity before symptoms arise. This may include advanced imaging technologies, biomarker detection, or other innovative methods. Additionally, future research needs to delve deeper into optimizing radiation doses, selecting targeted therapy drugs, and developing strategies for combining immunotherapy with radiation therapy, to better balance antitumor treatment and heart health protection, thus reducing the risk of cardiovascular complications.

## Author Contributions

**Dongmin Liu:** conceptualization, writing – original draft. **Yanling Yang:** writing – original draft. **Tingting Chen:** data curation. **Haonan Han:** data curation. **Yajing Yuan:** writing – review and editing. **Liming Xu:** writing – review and editing.

## Funding

This review was supported by the Tianjin Key Medical Discipline Construction Project TJYXZDXK‐3‐004B.

## Ethics Statement

The authors have nothing to report.

## Conflicts of Interest

The authors declare no conflicts of interest.

## Data Availability

Data sharing not applicable to this article as no datasets were generated or analysed during the current study.
